# Shear Bond Strength and Fracture Analysis of Human vs. Bovine Teeth

**DOI:** 10.1371/journal.pone.0059181

**Published:** 2013-03-18

**Authors:** Stefan Rüttermann, Anika Braun, Ralf Janda

**Affiliations:** Heinrich-Heine-University, Medical Faculty, Centre of Dentistry, Dept. of Operative Dentistry, Periodontology and Endodontology, Düsseldorf, Germany; University of Toronto, Canada

## Abstract

**Purpose:**

To evaluate if bovine enamel and dentin are appropriate substitutes for the respective human hard tooth tissues to test shear bond strength (SBS) and fracture analysis.

**Materials and Methods:**

80 sound and caries-free human erupted third molars and 80 freshly extracted bovine permanent central incisors (10 specimens for each group) were used to investigate enamel and dentine adhesion of one 2-step self-etch (SE) and one 3-step etch and rinse (E&R) product. To test SBS the buccal or labial areas were ground plane to obtain appropriate enamel or dentine areas. SE and E&R were applied and SBS was measured prior to and after 500 thermocycles between +5 and +55°C. Fracture analysis was performed for all debonded areas.

**Results:**

ANOVA revealed significant differences of enamel and dentin SBS prior to and after thermocycling for both of the adhesives. SBS- of E&R-bonded human enamel increased after thermocycling but SE-bonded did not. Bovine enamel SE-bonded showed higher SBS after TC but E&R-bonded had lower SBS. No differences were found for human dentin SE- or E&R-bonded prior to or after thermocycling but bovine dentin SE-bonded increased whereas bovine dentine E&R-bonded decreased. Considering the totalized and adhesive failures, fracture analysis did not show significances between the adhesives or the respective tooth tissues prior to or after thermocycling.

**Conclusion:**

Although SBS was different on human and bovine teeth, no differences were found for fracture analysis. This indicates that solely conducted SBS on bovine substrate are not sufficient to judge the perfomance of adhesives, thus bovine teeth are questionnable as a substrate for shear bond testing.

## Introduction

To harvest sound human teeth for in vitro testing of adhesive systems is becoming more and more difficult since indicated extractions are declining considerably. Furthermore ethical aspects have attracted more interest when human tissue is involved. Therefore, many scientists use bovine teeth as substitutes for human teeth to test bond strength [1]–[9]. As a consequence other authors have explored whether there are differences in bond strength [10]–[13], microleakage [14] and morphology [15], [16] of human versus bovine teeth. Camargo et al. [16] found that as regards the number of dentin tubules, the bovine specimens presented a significantly higher mean value than the human specimens but no difference in the diameters of human and bovine dentin tubules was observed. Bovine enamel was reported to demineralize and erode faster than human enamel [17]. Saleh et al. [10] and Schilke et al. [18] discovered highly significant differences between shear and tensile bond strengths of human and bovine enamel; however, regression prediction equations supported the use of bovine teeth as a reliable substitute to human counterparts in bonding studies of orthodontic adhesion [10].

Söderholm [19] stated in his letter to the editor that bond strength values do not present the true stress levels triggering failures of resin to hard tooth tissues adhesion. In his opinion taking a fracture mechanical approach might be more appropriate. It is widely accepted that shear bond test which pulls out tooth substrate must mean that the adhesive strength is superior to the cohesive strength of the tooth substrate, and that the meaning of the obtained value cannot be interpreted quantitatively anymore [20]. Following this hypothesis the present investigation did not only evaluate bovine as a substitute for human teeth by measuring shear bond strength but also by performing fracture analysis, which had not been done by the identified literature. The null hypothesis was that no differences between human and bovine teeth occur in (a) shear bond strength and (b) fracture analysis.

## Materials and Methods

Two commercial adhesives, one 2-step self-etch and one 3-step etch & rinse product, were selected (Table 1). Shear bond strength on human and bovine enamel and dentin prior to and after thermocycling was measured and fracture analysis was conducted after debonding.

**Table 1:** Test materials.

Material	Formulation	Manufacturer
Clearfil SE Bond: Primer #00590A, Adhesive#008 33 A	Primer: MDP, HEMA, hydrophilic aliphatic dimethacrylate, water, dyes, CQ; Adhesive (Bond): Bis-GMA, HEMA, hydrophobic aliphatic dimethacrylate, MDP, silanatedcolloidal silica, CQ, synergist: N,N-diethanol-p-toluidine	Kuraray Co. Inc., Kurashiki, Japan
Optibond FL: Primer#437388, Adhesive#437418	Etchant: 37,5% H_3_PO_4;_ Primer (Prime): HEMA, GPDM, MMEP, ethanol, water,initiator: CQ; Adhesive: Bis-GMA, TEGDMA, HEMA, GDMA, A174, fumed silica, bariumaluminoborosilicat glass. Na_2_SiF_6,_ YbF_3_, CQ, synergist: ODMAB,	Kerr Corp., Orange, CA, USA

A174 = 3-methacryloyloxypropyltrimethoxy silane, Bis-GMA = bisphenol-A-dimethacrylate, CQ = camphorquinone, GDMA = glycerol dimethacrylate, HEMA = 2-hydroxyethyl methacrylate, MDP = 10-methacryloyloxydecyl dihydrogenphosphate, ODMAB (synergist) = 2-(ethylhexyl)-4-(dimethylamino)benzoate, TEGDMA = triethylenglycol dimethacrylate

Formulations according to the respective material safety data sheet and the literature [42].

80 sound and caries-free human erupted third permanent molars of 18 to 40 year-old patients extracted for surgical reasons and 80 freshly extracted bovine permanent central incisors were thoroughly washed in running water and all blood and adherent tissues mechanically removed. The bovine teeth were not older than 2 days after the animals have been slaughtered. Regarding the human teeth all patients were informed, that their molars are used for scientific research. All patients gave their consent verbally. The samples were collected by two dentists in their private offices, collected and transferred to us anonymously, so that an identification of one individual tooth was impossible. The ethic committee of the medical faculty of the Heinrich-Heine-University of Düsseldorf gave formal approval (internal study number: 4094).

Until preparation for shear bond strength measurement, the teeth were stored no longer than a maximum period of 4 weeks according to ISO/TS 11405∶2003, the first week in a 0.5% chloramine T trihydrate (Sigma Aldrich Chemie GmbH, Taufkirchen, Germany) bacteriostatic/bactericidal solution and thereafter in distilled water at 4±2°C. To obtain similar dentin quality the teeth were X-rayed to determine the distance between the pulp chamber and the dentin surface to be bonded (X-ray device Philips Oralix U3-DC, Soredex Ltd., Helsinki, Finland, application data: 0.32 s, 10 mA, 60 kV, film Agfa Dentus M2, Class-D, Heraeus Kulzer GmbH, Hanau, Germany). Afterwards they were embedded in MMA/PMMA embedding resin (Technovit 4000, Heraeus Kulzer GmbH, Hanau, Germany) using a polyethylene mold (diameter: 25 mm, height: 30 mm) so that their buccal or labial areas, respectively, were close to the surface of the embedding resin. After removal from the mold, the teeth were ground under water cooling with 600 grit, 800 grit and finally 1000 grit grinding paper until a plane enamel or dentine area of at least 7 mm in diameter was exposed and a minimum dentin layer of 2.5 mm remained above the pulp chamber. Grinding was done by the same person by hand on a plane table.

The prepared human and bovine enamel and dentine specimens were randomly arranged in four groups of twenty specimens each for each of the adhesives, and their surfaces were treated with the respective adhesive according to the manufacturer’s instructions for use (Table 2). Thereafter a black opaque Teflon split mold (diameter: 25±0.5 mm, thickness: 2±0.1 mm) with a 3±0.1 mm diameter hole in its center was fixed on the thus treated dentine surfaces and the hole was filled with a first increment of approximately 0.8 mm with Clearfil AP-X (shade A3, #01122B, Kuraray Co. Inc., Kurashiki, Japan). The final increment was covered with a 0.05 µm transparent polyester foil prior to curing to avoid an inhibition layer. Each increment was light cured for 40 s. Light curing of all specimens was done with the tungsten halogen light Hilux Ultra Plus (Benlioglu Dental Inc. Ankara, Turkey) and the 11 mm diameter light guide in the constant polymerization mode (full light power from the start). Each time after a series of ten specimens had been cured the output of the curing device was checked with the Curing Light Meter (Benlioglu Dental Inc.). Irradiances between 750 and 850 mW cm^−2^ (mean 800±67 mW cm^−2^) were measured and no significant decrease of the output could be observed.

**Table 2:** Application protocol.

Material	Application
Clearfil SE Bond	Enamel or dentin was carefully dried with oil-free air. Primer was applied to entire tooth with a brush, left in place for 20 s and finally the volatile ingredients were evaporated for 10 to 15 s with a mild oil-free air stream. Bond was now also applied with a brush, dispersed with a very weak stream of air and polymerized for 10 s.
Optibond FL	Enamel or dentin was carefully dried with oil-free air. Etchant was applied on enamel for 30 s and on dentin for 15 s and then thoroughly rinsed off for 20 s with water. The tooth was gently dried with oil-free air to avoid desiccation. Prime (Bottle 1) was applied and rubbed in for 15 s and gently dried with air for approximately 5 s. Finally adhesive (Bottle 2) was applied, spread and with air to a thin layer and polymerized for 20 s.

**Figure 1:**
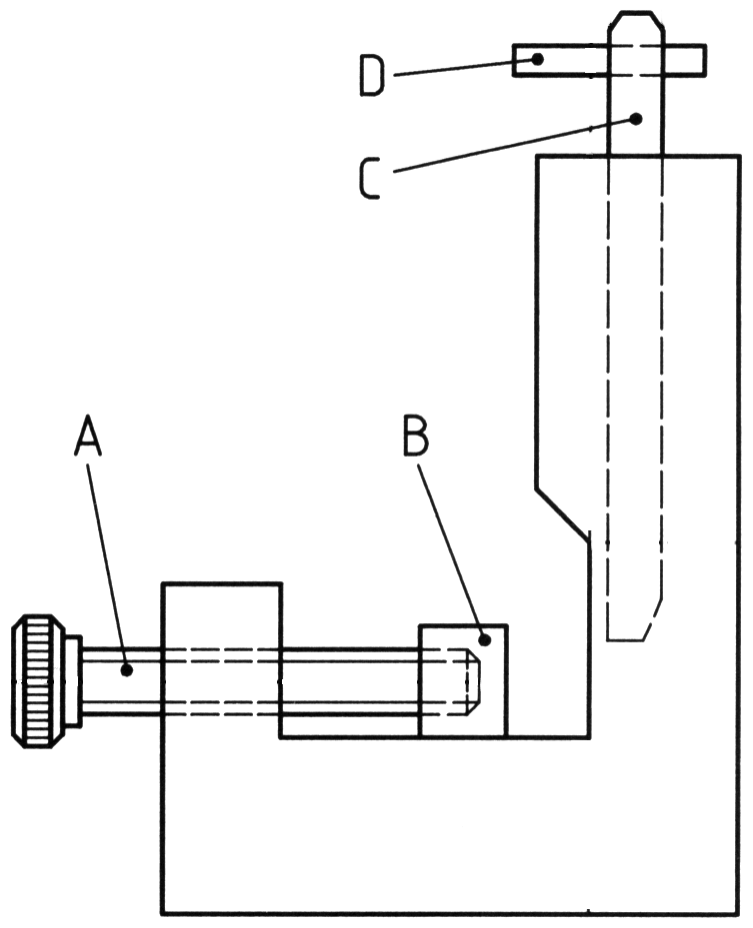
Shear bond strength device. A = fixation screw, B = fixation plate, C = plunger, D = stop pin.

After polymerization, all specimens were stored for 24 hours in water at 37°C. One half of the specimens was shear bond tested immediately and the other half was thermocycled 500 times in water between +5°C and +55°C. The specimens were left for 30 s at each temperature level. The transfer time was 15 s. The shear test was carried out according to ISO/TS 11405∶2003, Annex A test methods for measurement of bond strength [21] with a shear test device as described by ISO 10477 Amendment 1 (Figure 1) [22] and a Universal Testing Machine (Test GmbH, Erkrath, Germany). The cylinders formed by the resin-based restorative material had diameters of 3±0.1 mm and were loaded with a constant crosshead speed of 0.75 mm min^−1^. The load at break was recorded and the bond strength B was calculated in MPa using the formula B = F×S^−1^, in which F is the load in N at break and S is the bonded area of the cylinder in mm^2^.

Fracture analysis was conducted after the shear test. Digital photographs were taken from the de-bonded dentin surfaces (Canon EOS 20 D, Canon Inc., Tokyo, Japan) and fracture analysis was performed by visual inspection with Scion Image 4.0.2 scientific photo-software (Scion Corporation, Frederick, MD, USA).

### Statistical Analysis

Means and standard deviations were calculated. Normal distribution was tested by the Kolmogoroff-Smirnoff-Test. One-way ANOVA and post hoc Scheffé’s test were carried out for shear bond strength and surface tension (SPSS 15.0, SPSS, Chicago, IL, USA). This was performed separately for each of the different properties. Significant changes of shear bond strength prior to and after thermocycling were calculated with the lowest significant difference ANOVA. Results of the fracture analysis were compared with the non-parametric Mann-Whitney-U test. The Wilcoxon signed ranks test was used to calculate significances between the cohesive failures in the resin and the tooth for the respective material. Statistical significance for all tests was considered as p<0.05.

## Results

The results of the shear bond strength test are shown in Table 3 and the results of the fracture analysis are shown in Table 4.

**Table 3:** Shear bond strength, means and (standard deviations).

	Shear bond strength [MPa]							
	24 h storage at 37°C				500 thermocycles +5/+55°C			
	Enamel		Dentin		Enamel		Dentin	
	Human	Bovine	Human	Bovine	Human	Bovine	Human	Bovine
Clearfil SE Bond	21.1 (5.9)^a^	7.7 (2.8) ^abc^	11.7 (5.6)	12.6 (4.5)^e^	16.7 (6.0) ^c^	16.9 (4.1)^b^	15.4 (6.8)^d^	23.7 (4.8) ^de^
Optibond FL	23.0 (6.2)	24.1 (4.8)^d^	11.1 (4.9)^a^	19.8 (8.1)^ab^	25.4 (5.1)	18.2 (5.9)^d^	15.8 (5.7)^c^	8.9 (5.1) ^bc^

Significances of the respective hard tooth tissue within each row are indicated by superscript letters and are underlined within each column (p<0.05).

**Table 4:** Fracture analysis, means and (standard deviations).

			Fracture pattern in percent of bonded surface							
			24 h storage at 37°C				500 thermocycles +5/+55°C			
			Adhesive	Cohesive resin	Cohesive tooth	Cohesive total	Adhesive	Cohesive resin	Cohesive tooth	Cohesive total
Clearfil	Enamel	Human	24 (30)	*14 (22)* ^a^	*62 (29)* ^a^	76 (30)	12 (25)	*12 (14)*	*77 (24)*	89 (25)
SE Bond		Bovine	19 (31)	*28 (15)* ^a^	*54 (27)*	82 (31)	29 (44)	17 (24)	57 (40)	74 (43)
	Dentin	Human	16 (19)	28 (24)	57 (30 **)** ^b^	85 (19)	0 (0)	*21 (29* ***)***	*80 (29)*	100 (0)
		Bovine	22 (36)	*18 (16)*	*60 (36)*	78 (36)	10 (16)	*26 (19)*	*65 (31)*	91 (16)
Optibond	Enamel	Human	0 (0)	*7 (8)*	*93 (8)* ^a^	100 (0)	6 (19)	*12 (18)*	*82 (23)*	94 (19)
FL		Bovine	13 (22)	*12 (13)*	*75 (26)*	87 (22)	2 (6)	*24 (24)*	*75 (25)*	98 (6)
	Dentin	Human	0 (0)	*13 (12)* ^b^	*87 (12)* ^bc^	100 (0)	10 (32)	*12 (22)*	*78 (35)*	90 (32)
		Bovine	18 (25)	36 (23)^b^	46 (30)^c^	82 (25)	34 (36)	*9 (13)*	*57 (36)*	66 (36)

Significant differences (a) between human and bovine enamel or dentin, respectively, are indicated by the same superscript letter, (b) prior to and after TC are underlined, and (c) between cohesive failures in the resin and the tooth are in italic (p<0.05).

ANOVA revealed significant differences of enamel and dentin shear bond strength values prior to and after thermocycling for both of the tested adhesives. No differences were found for Clearfil SE Bond and Optibond FL human enamel specimens prior to and after thermocycling. The bovine enamel specimens of Clearfil SE Bond showed higher bond strength after thermocycling but the Optibond FL showed lower results. No differences were found for Clearfil SE Bond-treated or for Optibond FL-treated human dentin prior to and after thermocycling yet the bond strength of Clearfil SE Bond-treated bovine dentin specimens increased whereas that of the Optibond FL-treated specimens decreased.

Prior to and after thermocycling, significant differences in shear bond strength were found between human and bovine enamel as well for human and bovine dentin for both of the tested adhesives. Shear bond strengths of human and bovine enamel differed significantly for Clearfil SE Bond and Optibond FL prior to thermocycling, but after thermocycling the significance remained existent only for Optibond FL. The human and bovine dentin samples showed significant differences only for Optibond FL prior to but not after thermocycling; the Clearfil SE Bond samples behaved contrariwise.

Fracture analysis data did not reveal significances either between the materials or the respective hard tooth tissues when the adhesive and the totalized cohesive failures were considered. Significances were only detected in the cohesive failures in the resin or the tooth tissues, respectively. No correlation was found between shear bond strength and cohesive or adhesive failures, respectively.

## Discussion

To test adhesion on human enamel and dentin in comparison with bovine enamel and dentin, a conventional 3-step etch and rinse system and a 2-step self-etch system was used to evaluate if different results occurred for different bonding approaches. Shear bond strength measurement and fracture analysis are well established methods to evaluate resin enamel or resin dentin adhesion [12], [18], [21], [23]–[27]. Although the shear bond test is critically discussed and strongly competes with micro-tensile and micro-shear bond tests it is still considered to be a valid method and, therefore, is also used in the most recently published literature [25], [28]–[34]. However, also micro-tensile and micro-shear bond tests have to be discussed critically. Placido et al. [35] reviewed the different test methods and compared shear bond and micro-shear bond test using the finite element stress analysis. They concluded that although a shear load was applied for both tests, there was always a predominance of tensile stresses. They concluded further that the thicker relative adhesive layer in the micro-shear test concentrates stresses highly influencing the maximum load. Therefore, they judged micro-tensile test worse representing shear bond strength than shear test. There are also specific critical aspects for the micro-tensile test [20], [36]–[38]. Different values are achieved for different bonding areas meaning the smaller the area the higher the bond strength [35], [38] and the finite element analysis proved strong influence of specimen attachment and dimension on micro-tensile strength [36]. Therefore, there is no ideal bond test and there is still a need for the standardization of test procedures [20]. Thermocycling, an adequate procedure to simulate aging processes, is also required by ISO/TS 11405∶2003 “Dental materials - Testing of adhesion to tooth structure” [21]. There are numerous publications reporting bovine teeth being used to evaluate dentin bond strength of adhesive resins. But there are only few which directly compare the results obtained from bovine enamel or dentin with the respective human hard tooth tissues [11], [13], [18], [39].

Shear bond strength of standardized orthodontic brackets on human and bovine enamel was tested with the result that bond strength on bovine enamel was approximately 40% lower than on human enamel [40]. Statistical analysis from other authors also revealed a highly significant difference between shear and tensile bond strengths of human and bovine enamel; however, regression prediction equations supported the use of bovine teeth as a reliable substitute to human counterparts in bonding studies of orthodontic adhesion [10]. Reis et al. [11] used a micro tensile bond strength test to measure and compare bond strength of adhesive resins on human and bovine enamel and dentin. They found no statistically significant differences between these hard tooth tissues and concluded that bovine teeth proved to be possible substitutes for human teeth in either dentin or enamel bond testing, which is in accordance with other investigations [13]. The results of the present investigation (Tables 3 and 4) were in accordance with the literature. However, prior to thermocycling the 2-Step self-etch system Clearfil SE Bond performed significantly better on human than on bovine enamel but no significant difference was found for the 3-step etch and rinse adhesive Optibond FL. The opposite was observed after aging. Since bovine enamel and dentin develop more rapidly during tooth formation, bovine enamel has larger crystal grains and more lattice defects than human enamel [40]. There is a high probability that these facts influence bond strength because different grain sizes and defective lattice structures will be differently attacked by chemicals. This might explain the different performance of self-etch and etch and rinse adhesives.

The same unsteadiness is apparent in the results of the dentin measurements. Now Clearfil SE Bond (self-etch) showed no difference of shear bond strength between human and bovine dentin but Optibond FL (etch and rinse) performed better on bovine dentin. After thermocycling, Optibond FL lost but Clearfil SE Bond gained bond strength. Again, the findings of the present study is in accordance with some authors [12], [13] but not with other literature [11], [39]. Retief et al. [12] found significantly lower shear bond strength on bovine dentin despite more dense penetration of the adhesive system into bovine than into human dentin. Therefore, they concluded that the use of bovine teeth instead of human teeth is not indicated. It is quite certain that the different bond strength tests and materials used by the different authors caused the disagreements of the results for enamel as well as for dentin bond strength. However, also some morphological differences between human and bovine teeth have been reported [16], [41] that are due to the more rapid development of bovine enamel and dentin [40]. Furthermore, it has to be regarded that teeth are biological materials whose properties are influenced by various factors during formation and usage and therefore, vary in a broad range.

Adhesion was also investigated by microleakage studies. They showed that there are no statistically significant differences between the behavior of human and bovine substrates [14]. Also the fracture mechanical approach reported no statistical differences between human and bovine teeth but the authors admitted that there were a few exceptions [13]. The present study also performed the fracture analysis and evaluated cohesive failures in the tooth and the material as well as adhesive failures (Table 4). In nearly all cases significantly more cohesive failures occurred in the structures than in the materials indicating good bond strength. It is the authors’ opinion that only the totalized cohesive failures (cohesive in the tooth plus cohesive in the resin) are relevant to judge the adhesive quality because the adherence cannot be stronger than the inherent strength of the bonded materials. Therefore, no real bond strength values can be measured when cohesive fractures occur, which also explains why no correlation between shear bond strength and fracture pattern was detected in the present study. Furthermore, the fracture analysis results (Table 4) showed no significant differences either between human and bovine enamel or between human and bovine dentin prior to or after thermocycling for the adhesive, cohesive or totalized cohesive failures.

How can it be explained that sometimes shear bond strength was low but fracture analysis showed very high cohesive fracture rates? Shear bond strength is influenced by various factors as for instance, quality of the natural substrates, the conditioning method of the substrates’ surfaces, the aging method and/or the type of adhesive. Considering that bovine tooth structure has more lattice defects than human [40], acid etching (Optibond FL) might significantly weaken bovine teeth more than human teeth, resulting in lower bond strength after thermocycling. Acid etching might also attack bovine enamel more, yielding the same bond strength on human enamel with etch and rinse (Optibond FL) than with self-etch (Clearfil SE Bond) adhesives (Table 3). The literature supported the assumption that bovine enamel demineralizes and erodes faster than human enamel [17]. The aforesaid differences disappeared after thermocycling because bond strength increased for Clearfil SE Bond but decreased for Optibond FL. The authors hypothesize that stronger acid attack on bovine enamel and, therefore, stronger destruction became noticeable after thermocycling at lower bond strength values. The same reason might be of relevance when dentine shear bond strength values are considered. The self-etch product performed better on bovine dentine because it has minor destructive forces. None of these differences were reflected by the fracture analysis because neither of the adhesives differed in the failure rates but showed significantly more totalized cohesive than adhesive failures. This indicates a very good bond between all of the tooth tissues and the adhesives.

One major limitation of the present investigation is that only shear bond strength and no other bonding tests (i. e. microtensile bonding) were considered. Whereas some literature already called the shear bond strength test in question to be appropriate for bovine dentin [12] other authors performing a microtensile bond strength test did not report significant differences [11]. Furthermore, the authors cannot provide a relevant amount of totalized cohesive failures to judge an adhesive system to perform acceptably.

### Conclusion

There are numerous factors influencing bond strength between adhesives and tooth structures so that it is very difficult to interpret the results clearly. To obtain meaningful information about the performance of adhesives, fracture analysis is a condition sine qua non. Although shear bond strength was different on human and bovine teeth, no differences were found in fracture analysis. Therefore the null hypothesis was rejected for part (a) but accepted for part (b). Shear bond strength test on bovine teeth gives different quantitative results compared to human substrate, but additional fracture analysis on bovine teeth can give similar qualitative information. Thus, bovine teeth can only partly be recommended as a substitute for human teeth.
